# High-Fibre Breadsticks Fortified with Artichoke By-Product Extract and Inulin: Technological Quality, Phenolic Retention, and Consumer Acceptance

**DOI:** 10.3390/foods15213884

**Published:** 2026-10-29

**Authors:** Maria D’Elia, Fairouze Djeghim, Khawla Kerbab, Rita Celano, Valentina Santoro, Laura Smeraldo, Anna Lisa Piccinelli, Luca Rastrelli

**Affiliations:** 1 Department of Pharmacy, University of Salerno, Via Giovanni Paolo II, 132, 84084 Fisciano, Italy; mdelia@unisa.it (M.D.); rcelano@unisa.it (R.C.); vsantoro@unisa.it (V.S.); lsmeraldo@unisa.it (L.S.); apiccinelli@unisa.it (A.L.P.); 2 National Biodiversity Future Center—NBFC, 90133 Palermo, Italy; 3 Dipartimento di Scienze della Terra e Del Mare, University of Palermo, 90128 Palermo, Italy; 4 Institut de la Nutrition, de l’Alimentation et des Technologies Agro-Alimentaires (INATAA), Université Frères Mentouri Constantine 1, Constantine 25017, Algeria; fairouze.djeghim@umc.edu.dz; 5 Institut des Sciences et des Techniques Appliquées (ISTA) Ain M’Lila, Université of Oum El-Bouaghi, Oum El-Bouaghi 04000, Algeria; kerbab.khawla@univ-oeb.dz

**Keywords:** *Cynara cardunculus* L., fortified breadsticks, high-fibre bakery products, artichoke by-products, UHPLC-DAD-HRMS, antioxidant activity, sensory evaluation

## Abstract

High-fibre wheat breadsticks were developed using an artichoke (*Cynara cardunculus* L.) by-product extract standardized to 5% chlorogenic acid, incorporated at 1.5%, 2.5%, and 3.5% (*w*/*w*, flour basis), together with 5% purified artichoke-derived inulin. A non-fortified formulation was used as the control. Technological, nutritional, textural, colour, sensory, phytochemical, and antioxidant properties were evaluated. Based on technological performance and preliminary sensory screening in 30 participants, the formulation containing 2.5% artichoke extract was selected as the most suitable compromise. Total dietary fibre increased from 4.08 g/100 g in the control to approximately 8.8 g/100 g in the fortified breadsticks, while the selected formulation contained 4.92 ± 0.21 g/100 g of fructans. UHPLC-DAD-HRMS/MS analysis confirmed the persistence of characteristic artichoke-derived caffeoylquinic acids and luteolin derivatives throughout processing. The selected breadsticks contained 83.30 ± 1.99 mg/100 g of total quantified phenolics, including 79.17 ± 2.07 mg/100 g of caffeoylquinic acid derivatives. After correction for the 30.48% baking mass loss, the overall retention of the eight quantified phenolic markers was 81.5%, with 81.8% retention of caffeoylquinic acid derivatives. The final product also showed measurable ABTS radical-scavenging activity and favorable sensory liking, receiving an overall score of 4.50 ± 0.51 from 50 consumers. These findings support the combined use of standardized phenolic and inulin fractions recovered from artichoke by-products for the development of nutritionally enhanced bakery products.

## 1. Introduction

Bakery products are widely consumed and represent suitable matrices for nutritional and functional reformulation because of their familiarity, convenience, and versatility. Breadsticks, in particular, combine a crisp texture and relatively low moisture content with the possibility of incorporating dietary fibre, plant-derived compounds, and ingredients recovered from agro-industrial by-products. A recent systematic review of fortified breadsticks and related bakery products showed that cereal fractions, plant powders, extracts, and processing by-products can increase dietary fibre, phenolic compounds, micronutrients, and antioxidant capacity. However, considerable variability in formulations, processing conditions, analytical methods, and sensory protocols indicates that nutritional improvement must be evaluated together with technological performance and consumer acceptance [1].

The incorporation of plant-derived ingredients into bakery matrices may modify water absorption, dough consistency, product expansion, texture, colour, flavour, and overall acceptability. These effects depend largely on the composition and concentration of the added ingredient and on its interactions with the cereal matrix [1,2]. In breadsticks fortified with red grape pomace, Rainero et al. reported increases in dietary fibre, phenolic content, and antioxidant capacity, together with marked changes in dough properties, volume, hardness, colour, and sensory perception [3]. Likewise, the incorporation of *Wolffia globosa* powder improved the nutritional and functional characteristics of breadsticks, although the highest inclusion level negatively affected sensory acceptability [4]. Similarly, fortification of wheat bread with *Matricaria chamomilla* enhanced fibre content and antioxidant properties, while the form and concentration of the plant ingredient influenced technological and sensory characteristics [5]. Collectively, these findings indicate that increasing the level of a functional ingredient does not necessarily result in the most suitable product and highlight the need to integrate nutritional, technological, and sensory evaluation during formulation development.

The valorization of fruit and vegetable processing by-products is particularly relevant in this context. Peels, stems, leaves, bracts, and pomaces may retain dietary fibre and phytochemicals that can be recovered and incorporated into new food products [6]. Their use can enhance the nutritional and functional value of bakery products while supporting circular-economy strategies and reducing the amount of plant biomass directed to disposal. Nevertheless, by-product-derived ingredients may also modify gluten development, water distribution, browning reactions, mechanical properties, and sensory characteristics. Their successful application therefore depends on the recovery process, ingredient composition, incorporation level, and processing conditions [2].

Globe artichoke processing generates substantial quantities of leaves, stems, and external bracts that retain valuable phenolic compounds and fructans. These by-products contain mono- and dicaffeoylquinic acids, including chlorogenic acid and cynarin, together with luteolin and apigenin derivatives [7,8]. They are also a source of inulin-type fructans, which have been recovered from both stems and bracts through processes designed to support the integrated valorization of the artichoke supply chain [9,10].

Quantitative data further support their nutritional and functional potential. Cannas et al. reported total dietary fibre contents of 68.57 and 43.63 g/100 g dry matter in artichoke outer-bract and stem powders, respectively [11]. When incorporated into breadsticks at 5%, these powders increased dietary fibre from 5.00 g/100 g dry matter in the control to 7.59 g/100 g with outer bracts and 6.52 g/100 g with stems. The 5% stem-fortified breadsticks also showed increases of 527% and 114% in DPPH and ABTS radical- scavenging capacity, respectively, compared with the control [11]. Green extraction approaches, including pressurized hot-water extraction, have been optimized for the recovery of caffeoylquinic acids and flavonoids from artichoke residues, providing high extraction yields while limiting the formation of thermally induced artefacts [12,13].

The recovery of phenolic compounds and inulin from the same artichoke biomass offers two complementary ingredients for bakery fortification. Purified artichoke-derived inulin can contribute dietary fibre, whereas the phenolic extract provides characteristic artichoke metabolites and antioxidant capacity. However, both ingredients may affect dough hydration, texture, colour development, and sensory perception, making careful selection of their incorporation levels essential.

Although artichoke by-products have been investigated as sources of phenolic compounds and inulin, limited information is available on the simultaneous incorporation of separately recovered and standardized phenolic and inulin fractions into breadsticks. In particular, it remains unclear how such a combined formulation strategy influences dough and baked-product technological properties, sensory acceptability, and the retention of characteristic artichoke-derived phenolic markers during baking. Moreover, the quantitative fate of these phenolic markers throughout breadstick processing has been scarcely investigated. An integrated evaluation combining technological, nutritional, sensory, and high-resolution phytochemical analyses, together with assessment of phenolic retention during baking, may therefore provide a more comprehensive evaluation of their suitability as functional bakery ingredients. Previous artichoke-fortified bakery studies have mainly focused on whole by-product powders or phenolic-rich extracts used as individual fortifying ingredients. In contrast, the present study combines a standardized phenolic extract with purified inulin separately recovered from the same artichoke biomass and quantitatively follows characteristic phenolic markers from the initial extract through dough processing and baking. This combined fractionation-based approach, together with mass-corrected assessment of phenolic retention, distinguishes the present work from previous artichoke-fortification studies.

In the present study, wheat breadsticks were fortified with an artichoke by-product extract standardized to 5% chlorogenic acid at 1.5%, 2.5%, and 3.5% on a flour-weight basis, while purified artichoke-derived inulin was maintained at 5% in all fortified formulations. The inulin level was kept constant to provide a fixed fibre-enrichment background while allowing the effect of increasing concentrations of the standardized phenolic extract to be evaluated. Accordingly, the experimental design was intended to identify the most suitable extract level among those tested. The primary objective was to identify the most suitable extract concentration by integrating physical and baking properties, dough texture, maximum breadstick cutting force, crust and crumb colour, nutritional composition, and preliminary sensory acceptability. We hypothesized that the combined use of the two separately recovered artichoke-derived fractions would increase fibre content and provide measurable retention of characteristic phenolic markers during processing while maintaining acceptable technological and sensory properties. The selected formulation was further characterized by UHPLC-DAD-HRMS/MS profiling and quantitative analysis of the initial extract, fortified dough, and baked breadsticks, together with assessment of mass-corrected phenolic retention, antioxidant activity, fructan content, and consumer acceptance. The expected scientific contribution was to provide an integrated evaluation of a fractionation-based artichoke by-product valorization strategy for bakery fortification, linking formulation performance with quantitative tracking of characteristic phenolic compounds during processing.

## 2. Materials and Methods

### 2.1. Reagents and Materials

Food-grade germ-enriched soft wheat flour, refined type 00 wheat flour, instant dry yeast, olive oil, and sodium chloride were obtained from certified local suppliers in Constantine, Algeria, and used without further treatment. The artichoke by-product extract, standardized to 5% chlorogenic acid, and the purified artichoke-derived inulin were obtained from by-products of a selected C3 G1 artichoke (*Cynara cardunculus* L.) cultivar and were prepared as described in Section 2.2 and Section 2.3. Potable water was used for breadstick preparation. Analytical-grade ethanol and methanol were purchased from Merck Chemicals (Milan, Italy). LC-MS-grade acetonitrile, water, and formic acid were purchased from Romil Ltd. (Cambridge, UK). Ultrapure water (18.2 MΩ·cm) was produced using a Milli-Q water purification system (Millipore, Bedford, MA, USA). Reference standards of chlorogenic acid (purity ≥ 98%; Extrasynthese, Lyon, France), luteolin-7-*O*-glucoside (purity 98%; Sigma-Aldrich, St. Louis, MO, USA), and cynarin (purity 98%; Sigma-Aldrich, St. Louis, MO, USA) were used for chromatographic identification and quantification. For the ABTS assay, 2,2′-azinobis(3-ethylbenzothiazoline-6-sulfonic acid), potassium persulfate, and Trolox were obtained from Merck Chemicals (Milan, Italy). The K-FRUC Fructan Assay Kit was purchased from Megazyme (Bray, Ireland).

### 2.2. Preparation and Standardization of the Artichoke By-Product Extract

Artichoke processing by-products, consisting mainly of leaves and stems, were air-dried, milled, and subjected to aqueous ultrasound-assisted extraction. Water was used as the sole extraction solvent at a solid-to-liquid ratio of 1:20 (*w*/*v*). Extraction was carried out at 60 °C using an ultrasonic system operating at 20 kHz for three consecutive extraction cycles of 30 min each. After each extraction cycle, the aqueous phase was separated from the insoluble plant material, and the three extracts were pooled. The combined aqueous extract was concentrated under reduced pressure and spray-dried to obtain a stable powder. The extraction conditions were selected on the basis of previous laboratory experience with the recovery of phenolic compounds from artichoke by-products [12,13]. The resulting artichoke by-product extract was standardized to 5% chlorogenic acid by chromatographic analysis and stored at −20 °C in sealed containers protected from light and moisture until use. A single production batch was used for all breadstick formulations and analytical determinations.

### 2.3. Preparation of Artichoke-Derived Inulin

Artichoke-derived inulin was prepared from the same artichoke processing by-products used for phenolic extraction. The aqueous extract obtained from the plant material was clarified to remove insoluble residues, and the carbohydrate-rich fraction was isolated according to previously reported procedures for the recovery of inulin-type fructans from artichoke by-products [9,10]. The fraction was concentrated and dried to obtain a stable powder suitable for food formulation. The fructan content of the resulting powder was determined enzymatically according to the method described in Section 2.10. The powder was stored at −20 °C in sealed containers protected from moisture until use. A single production batch was used throughout the study and was incorporated into all fortified breadstick formulations at a fixed level of 5% on a flour-weight basis.

### 2.4. Dough Formulation and Breadstick Preparation

Breadsticks were prepared using a wheat flour blend consisting of 800 g germ-enriched soft wheat flour and 200 g refined type 00 wheat flour. Each formulation also contained 50 g instant dry yeast, 20 g sodium chloride, and 20 g olive oil. Three fortified formulations were prepared by adding 15, 25, or 35 g of standardized artichoke by-product extract per kilogram of total flour, corresponding to 1.5%, 2.5%, and 3.5% (*w*/*w*, flour basis), respectively. Purified artichoke-derived inulin was added to all fortified formulations at 50 g/kg flour (5%, *w*/*w*), whereas the control contained neither artichoke extract nor inulin. The dry ingredients were initially blended manually with a spatula for 1 min to ensure uniform distribution. Water and olive oil were then added, and the dough was mechanically kneaded using a laboratory-scale dough mixer for 15 min (Kenwood, Hampshire, UK). The amount of water was adjusted empirically for each formulation during preliminary trials to obtain a homogeneous and workable dough suitable for breadstick shaping. No farinograph, solvent retention capacity, or other instrumental hydration measurements were used to standardize water addition. The exact amounts used were 780 mL for the control, 680 mL for the 1.5% formulation, 672 mL for the 2.5% formulation, and 690 mL for the 3.5% formulation. Following kneading, the dough was subjected to a first fermentation step for 30 min at 30 °C. It was then divided and manually shaped into individual breadsticks weighing 16.5 g and measuring approximately 15 cm in length. The shaped pieces underwent a second fermentation step for 30 min at 30 °C and were subsequently baked in a preheated oven at 180 °C for 25 min. Immediately after removal from the oven, each breadstick was weighed for the calculation of baking weight loss. The breadsticks were then cooled to room temperature and stored in airtight containers until analysis.

### 2.5. Selection of the Preferred Formulation

Four breadstick formulations were prepared: a non-fortified control and three fortified formulations containing 1.5%, 2.5%, and 3.5% standardized artichoke by-product extract on a flour-weight basis. Artichoke-derived inulin was incorporated at a fixed concentration of 5% in all fortified formulations, whereas the control contained neither artichoke extract nor inulin.

The experimental study was conducted in two consecutive stages. During the preliminary stage, the control and the three fortified formulations were comparatively evaluated for their physical and baking properties, dough texture, breadstick cutting force, crust and crumb color, and sensory acceptability. The same group of 30 participants evaluated all four formulations using a 9-point hedonic scale for color, taste, and overall acceptability. Participants were subsequently asked to select their preferred enriched formulation among the 1.5%, 2.5%, and 3.5% samples.

The formulation containing 2.5% artichoke extract was selected as the most suitable compromise between extract incorporation, technological performance, and preliminary sensory acceptability. Nutritional composition was determined for the control and all three fortified formulations.

During the second stage, the selected 2.5% formulation underwent detailed phytochemical and antioxidant characterization and was evaluated by an independent group of 50 consumers. UHPLC-DAD-HRMS/MS analysis was performed on the initial artichoke by-product extract, the control dough, the fortified dough before baking, the control breadsticks, and the corresponding fortified breadsticks after baking. Qualitative and quantitative phenolic profiling, including assessment of phenolic retention during baking, together with antioxidant activity and fructan content, was performed according to the analytical procedures described in Section 2.7, Section 2.8, Section 2.9 and Section 2.10. Because the control contained neither artichoke extract nor inulin, comparisons between the control and fortified formulations represent the combined effect of the two artichoke-derived ingredients. Comparisons among the three fortified formulations primarily reflect the effect of increasing extract concentration, since the inulin level was maintained constant.

### 2.6. Preparation of Samples for Phytochemical and Antioxidant Analyses

The initial artichoke by-product extract, control dough, dough fortified with 2.5% artichoke extract, control breadsticks, and breadsticks fortified with 2.5% artichoke extract were subjected to phytochemical analysis and antioxidant activity assays. Samples were extracted in triplicate by ultrasound-assisted solid–liquid extraction using an ultrasonic bath (Bandelin Electronic, Berlin, Germany). Each sample was subjected to three consecutive extraction cycles of 30 min at 25 °C using 70% (*v*/*v*) aqueous ethanol at a sample-to-solvent ratio of 1:20 (*w*/*v*). At the end of each extraction cycle, the samples were centrifuged at 13,000× *g* for 10 min using a Sorvall ST 16 centrifuge (Thermo Fisher Scientific, Milan, Italy). The supernatants were collected, filtered through Whatman No. 1 filter paper, and pooled. The exhaustiveness of the extraction was verified by chromatographic monitoring of each consecutive extraction cycle; in the third cycle, the areas of the main peaks were <2% of the corresponding cumulative peak areas. Ethanol was removed under reduced pressure at 40 °C using a Rotavapor R-200 rotary evaporator (Büchi Italia S.r.l., Cornaredo, Italy). The remaining aqueous fractions were subsequently freeze-dried to obtain the exhaustive dry extracts used for chromatographic and antioxidant analyses. Aliquots of the freeze-dried extracts intended for the ABTS assay were dissolved in methanol at a concentration of 1 mg mL^−1^ and appropriately diluted with 5 mM phosphate-buffered saline (PBS, pH 7.4) before analysis. For UHPLC-DAD-HRMS/MS analysis, the freeze-dried extracts were reconstituted in methanol at a concentration of 10 mg mL^−1^ before injection.

### 2.7. Identification of Metabolites by UHPLC-DAD-HRMS/MS

Metabolite identification was performed using a Vanquish Flex UHPLC system (Thermo Fisher Scientific, Milan, Italy) coupled in series to a diode array detector (DAD) and an Orbitrap Exploris 120 mass spectrometer (Thermo Fisher Scientific, Milan, Italy) equipped with a heated electrospray ionization (HESI-II) source operating in negative ionization mode.

Chromatographic separation was carried out on a Kinetex PFP column (100 × 2.1 mm, 2.6 μm particle size; Phenomenex, Torrance, CA, USA) operated at 30 °C with a flow rate of 400 μL min^−1^ and a binary gradient of water (A) and MeCN (B), both containing 0.1% formic acid. The injection volume was 5 μL. The elution method was as follows: 5–10% B (0–2 min), 10% B (2–7 min), 10–15% B (7–12 min), 15–24% B (12–21 min), 24–98% B (21–24 min), 98% B (24–26 min), 98–5% B (26–27 min), followed by re-equilibration at 5% B until 31 min.

The UV spectra were acquired in the range of 200–600 nm, and HRMS data were acquired in negative ionization mode. A Full MS data-dependent MS/MS (Full MS/dd-MS2) acquisition mode was used, with the resolution of Full MS scan (scan range *m*/*z* 100–1000) and dd-MS2 scan of 60,000 and 30,000 FWHM, respectively. A stepped collision energy HCD (20, 40, and 60) was applied for fragmentation. Instrument control and spectra acquisition were carried out using Xcalibur software version 4.6 (ThermoFisher Scientific).

Compound annotation was achieved by combining accurate mass measurements, molecular formula prediction, MS/MS fragmentation patterns, and UV–Vis spectral data. The confidence level of metabolite identification was assigned according to the Metabolomics Standards Initiative (MSI) guidelines: Level 1, confirmed identification based on comparison with authentic reference standards; Level 2, putatively annotated compounds based on MS/MS spectral matching with literature or spectral databases; and Level 3, putatively characterized compound classes based on diagnostic fragmentation patterns and chemotaxonomic evidence.

### 2.8. UHPLC–DAD Quantitative Analysis and Calculation of Phenolic Retention

Quantitative analysis was performed under the same chromatographic separation conditions described in Section 2.7, using UHPLC-DAD detection and an external calibration method with authentic reference standards. Individual stock solutions of chlorogenic acid and cynarin were prepared in ethanol at a concentration of 1 mg mL^−1^, whereas luteolin-7-*O*-glucoside was dissolved in dimethyl sulfoxide (DMSO) at the same concentration. All stock solutions were protected from light and stored at 4 °C until use.

A mixed standard solution containing the three reference compounds was prepared in methanol at a concentration of 100 μg mL^−1^ for each analyte. Calibration standards were subsequently obtained by serial dilution of this solution, generating eight concentration levels (1, 5, 10, 20, 50, 80, 100, and 150 μg mL^−1^). External calibration curves were constructed over these concentration ranges, and a linear regression model was applied for quantitative determination. The linearity of the model was verified by ANOVA, with R^2^ values exceeding 0.999, and the resulting calibration ranges are reported in Table 1. Intraday precision was evaluated at all concentration levels, with coefficients of variation (CV) below 5%, confirming good repeatability of the analytical method.

Two wavelengths were selected for the detection of target analytes based on their absorption maxima: 325 nm for caffeoylquinic acid derivatives and 350 nm for luteolin derivatives. Mono-caffeoylquinic acid derivatives (compounds A–C) were quantified as chlorogenic acid equivalents. Cynarin and the other dicaffeoylquinic acid derivatives (compounds D, G, and H) were quantified as cynarin equivalents, whereas luteolin derivatives (compounds E and F) were quantified as luteolin-7-*O*-glucoside equivalents. Quantitative results for the initial artichoke by-product extract were expressed as mg/g of dry extract, whereas concentrations in the fortified dough and baked breadsticks were expressed as mg/100 g on an as-is basis. All determinations were performed in triplicate.

For the selected 2.5% formulation, the retention of individual phenolic compounds during baking was calculated on a mass basis by correcting the concentration measured in the baked breadsticks for the experimentally determined baking mass loss (Equation (1))

Baking retention (%) = [(C_baked_ × M_baked_)/(C_dough_ × M_dough_)] × 100 (1)

where C_dough_ and C_baked_ are the concentrations of the compound in the fortified dough and baked breadsticks, respectively, and M_dough_ and M_baked_ are the corresponding sample masses before and after baking. The M_baked_/M_dough_ ratio was obtained from the experimentally determined baking weight loss described in Section 2.11. Retention percentages were calculated from the mean quantitative concentrations and the mean baking mass loss of the selected formulation. The sum of caffeoylquinic acid derivatives included compounds A–D and G–H, whereas the sum of luteolin derivatives included compounds E and F. Total quantified phenolics corresponded to the sum of all eight compounds.

### 2.9. Determination of Antioxidant Activity

The antioxidant capacity of the breadsticks was evaluated using ABTS scavenging capacity according to Celano et al. [14]. Briefly, 10 μL of sample, Trolox standard solution, or 5 mM phosphate-buffered saline (PBS; blank), appropriately diluted in 5 mM PBS, was mixed with 1 mL of ABTS•^+^ working solution (approximately 0.1 mM). Aliquots (300 μL) of each reaction mixture were transferred to a 96-well microplate, and the absorbance was measured at 734 nm after 45 min of incubation at 30 °C using a Varioskan LUX microplate reader (Thermo Fisher Scientific). Trolox was used as the reference antioxidant for calibration. A six-point calibration curve (0.25–2.5 mM) was prepared by serial dilution of a 2.5 mM Trolox stock solution. All measurements were performed in triplicate. Antioxidant activity was expressed as Trolox equivalent antioxidant capacity and reported as µmol Trolox equivalents per gram of breadsticks on an as-is basis.

### 2.10. Determination of Fructan Content

The inulin (fructan) content of breadsticks was determined using AOAC Official Method 999.03 and the K-FRUC Fructan Assay Kit (Megazyme, Bray, Ireland) according to the manufacturer’s instructions [15]. Briefly, 400 mg of finely ground sample was suspended in 25 mL of distilled water and incubated in a boiling water bath for 10 min, with agitation after 5 min. After cooling to room temperature, 2.0 mL of the extract was transferred into a microcentrifuge tube and centrifuged at 13,000 rpm. An aliquot (0.2 mL) of the resulting supernatant was then subjected to the enzymatic assay.

Aliquots (0.2 mL) of the extract were incubated with 0.2 mL of diluted sucrase/β-amylase/pullulanase/maltase solution at 40 °C for 30 min to hydrolyse sucrose and digest starch-derived carbohydrates. Subsequently, 0.2 mL of alkaline borohydride solution (10 mg mL^−1^ sodium borohydride in 50 mM sodium hydroxide) was added, and the mixture was incubated at 40 °C for a further 30 min to reduce free reducing sugars. Excess borohydride was neutralized and the pH adjusted to approximately 4.5 by adding 0.5 mL of 200 mM acetic acid. Fructans were hydrolysed by adding 0.1 mL of fructanase solution and incubating the samples at 40 °C for 30 min. Corresponding blanks were prepared by replacing the enzyme solution with 0.1 M sodium acetate buffer. After enzymatic hydrolysis, 5.0 mL of PAHBAH working reagent was added, and the tubes were heated in a boiling-water bath for exactly 6 min. The tubes were immediately cooled in water at 18–20 °C for 5 min, and absorbance was measured at 410 nm against the reagent blank. Fructan content was calculated according to the K-FRUC assay procedure and expressed as g of fructans (as inulin) per 100 g of breadsticks on an as-is basis. Measurements were performed in triplicate.

### 2.11. Physical and Baking Properties of Breadsticks

The physical and baking properties of the control and fortified breadsticks were evaluated by determining volume, specific volume, baking weight loss, and moisture content. Breadstick volume was determined after cooling to room temperature using the rapeseed displacement method according to AACC Method 10-05 [16]. Specific volume was calculated as the ratio between breadstick volume and the corresponding baked-product weight and expressed as cm^3^/g (Equation (2)).

(2) $$\mathrm{Specific volume}=\frac{{\mathrm{Breadstick volume}\;(\mathrm{cm}}^{3})}{\mathrm{Breadstick weight}\;(g)}$$

Baking weight loss was calculated using the mass of each shaped dough piece and fixed at 16.5 g before the second fermentation, and the mass of the corresponding breadstick was measured immediately after removal from the oven (Equation (3)):

(3) $$\mathrm{Baking weight loss}\left(\%\right)=\frac{W_{\mathrm{dough}}-W_{\mathrm{breadstick}}}{W_{\mathrm{dough}}}\times 100$$

where *W*_dough_ is the mass of the shaped dough piece before the second fermentation and *W*_breadstick_ is the mass of the corresponding breadstick immediately after baking. Breadstick moisture content was determined according to ICC Standard Method 110/1 [17] and expressed as g/100 g of product (% *w*/*w*). For each formulation, measurements were performed in triplicate on samples obtained from a single baking batch.

### 2.12. Dough Texture Analysis by Back Extrusion

The textural properties of the doughs were evaluated immediately after the second fermentation step using a two-cycle back-extrusion test, following the procedure described by Uribe-Wandurraga et al. [18], with minor adaptations. Measurements were performed using a Shimadzu EZ-SX texture analyzer (Shimadzu Corporation, Kyoto, Japan) equipped with a 500 N load cell. For each measurement, 60 g of dough was transferred into a cylindrical glass container with an internal diameter of 50 mm. A cylindrical probe with a diameter of 30 mm was positioned centrally above the sample. The dough was subjected to two consecutive compression cycles using pre-test and test speeds of 1.0 mm/s and a post-test speed of 10 mm/s. Hardness, adhesiveness, springiness, cohesiveness, gumminess, and chewiness were obtained from the resulting force–time curves using the instrument software. The test was conducted under the same conditions for the control and all fortified formulations. Five replicate measurements were performed for each formulation using dough obtained from the same production batch. These measurements therefore represent technical replicates rather than independent production replicates.

### 2.13. Breadstick Texture Analysis

The mechanical properties of the baked breadsticks were evaluated using a cutting test adapted from Uribe-Wandurraga et al. [18] and performed with the same Shimadzu EZ-SX texture analyzer equipped with a 500 N load cell. Five breadsticks were randomly selected from each formulation and analysed after cooling to room temperature. These breadsticks represented within-batch technical replicates from the same production batch. Each breadstick was positioned horizontally on the instrument platform and cut at its midpoint using a flat-ended toothed pushrod A. The probe travelled downward for 12 mm at a constant speed of 3.0 mm/s during the pre-test, test, and post-test phases before returning to its initial position. Maximum cutting force was expressed in newtons as the highest force required to cut the sample.

### 2.14. Crust and Internal Crumb Color

The color of the breadstick crust and internal crumb was determined in the CIELAB color space using the Color Grab Android application (version 3.6.1; Loomatix), following the approach described by Djeghim et al. [19]. To minimize variation caused by ambient light, measurements were conducted inside a closed polystyrene chamber with white internal walls and a 1.2 W, 5 V white LED light source. Lighting conditions, camera position, and sample distance were kept constant throughout the measurements. Before each measurement session, the system was calibrated using a standard white reference placed inside the chamber under the same fixed acquisition conditions. Color was expressed as L* (lightness, from 0 = black to 100 = white), a* (green–red axis), and b* (blue–yellow axis). The total color difference (ΔE*) between the control and each fortified formulation was calculated separately for the crust and internal crumb using Equation (4):

(4) $$\Delta E^{*}=\sqrt{{\left(L_{s}^{*}-L_{c}^{*}\right)}^{2}+{\left(a_{s}^{*}-a_{c}^{*}\right)}^{2}+{\left(b_{s}^{*}-b_{c}^{*}\right)}^{2}}$$

where $L_{c}^{*}$, $a_{c}^{*}$ and $b_{c}^{*}$ are the coordinates of the control breadsticks, while $L_{s}^{*}$, $a_{s}^{*}$ and $b_{s}^{*}$ are the corresponding values of the fortified samples. For each formulation, crust and crumb colour parameters were measured in five technical replicates on breadsticks obtained from the same production batch.

### 2.15. Nutritional Composition and Energy Value

The nutritional composition of the control and fortified breadsticks was determined on the final baked products using official AOAC methods [20]. Moisture, protein, total lipid, ash, and total dietary fibre contents were determined in analytical triplicate on samples obtained from a single baking batch for each formulation and expressed as g/100 g of breadsticks on an as-is basis. Moisture content was determined according to ICC Standard Method 110/1. Protein content was determined using the Kjeldahl method according to AOAC Method 920.87, applying a nitrogen-to-protein conversion factor of 6.25. Total lipid content was determined by hot extraction with petroleum ether according to AOAC Method 945.16. Ash content was determined by incineration at 550 °C according to AOAC Method 923.03. Total dietary fibre was measured using the enzymatic–gravimetric procedure described in AOAC Method 991.43. Carbohydrate content was calculated by difference for each analytical replicate. The energy value was calculated from the mean compositional values using conversion factors of 4 kcal/g for proteins and carbohydrates, 9 kcal/g for lipids, and 2 kcal/g for total dietary fibre. Energy values in kilojoules were obtained using the conversion factor 1 kcal = 4.184 kJ. Experimentally determined parameters and calculated carbohydrate values were reported as mean ± standard deviation of three determinations, whereas calculated energy values were reported without standard deviations.

### 2.16. Preliminary Sensory Evaluation Protocol

The control and the three fortified breadstick formulations containing 1.5%, 2.5%, and 3.5% artichoke by-product extract were evaluated by the same group of 30 participants. Samples were identified by randomized, three-digit codes and presented in randomized order. Color, taste, and overall acceptability were rated using a 9-point hedonic scale ranging from 1 (“dislike extremely”) to 9 (“like extremely”), allowing greater discrimination among formulations during the preliminary screening. Water was provided for palate cleansing between samples. After evaluating all four formulations, participants were asked to indicate their single preferred enriched formulation among the 1.5%, 2.5%, and 3.5% samples. The technological and preliminary sensory results were jointly considered when selecting the preferred fortified formulation. The questionnaire used for the preliminary sensory screening is provided as Questionnaire S1.

### 2.17. Consumer Acceptance Test of the Selected Formulation

Following the preliminary screening, the selected breadstick formulation containing 2.5% artichoke extract and 5% inulin was evaluated by an independent group of 50 consumers. Consumer acceptance of the selected formulation was assessed using a simpler 5-point scale ranging from 1 (“very unsatisfactory”) to 5 (“very satisfactory”) to minimize cognitive load and evaluation fatigue for untrained panelists. Consumer acceptance was assessed across appearance and presentation, breadstick size, aroma, crispness, taste, aftertaste, ease of chewing, and overall liking. Individual questionnaire scores were used to calculate descriptive statistics and score-frequency distributions for each attribute. Participation in both sensory evaluations was voluntary, and responses were collected using anonymous participant codes. The consumer acceptance questionnaire is provided as Questionnaire S2.

### 2.18. Statistical Analysis

Statistical analyses were performed using JMP Pro 17.0.0 (SAS Institute Inc., Cary, NC, USA). One dough preparation and baking batch was produced for each formulation. Technological and physicochemical measurements were performed as technical replicates on samples obtained from the same production batch, with the number of replicate measurements specified in the corresponding methodological subsection. These measurements therefore do not represent independent production replicates. Nutritional composition, quantitative phenolic analysis, antioxidant activity, and fructan content were likewise determined using analytical replicates and are reported as mean ± standard deviation. Because independent production batches were not available, inferential statistical comparisons among formulations were not performed for technological, physicochemical, or compositional measurements, and these data were evaluated descriptively. Baking-retention percentages were calculated from mean quantitative concentrations after correction for the experimentally determined baking mass loss and were also reported descriptively. Energy values were calculated from mean compositional data and reported without standard deviations. Preliminary sensory scores from the same 30 participants evaluating all four formulations were analysed as repeated measures using the Friedman test, followed, when significant, by Wilcoxon signed-rank tests with Holm adjustment. Preferred enriched-formulation frequencies were assessed using a chi-square goodness-of-fit test against an equal expected distribution. Consumer-acceptance data for the selected 2.5% formulation (*n* = 50) were analysed descriptively using mean ± standard deviation, median and interquartile range, score-frequency distributions, and the percentage of positive ratings (scores of 4 or 5). An exploratory principal component analysis (PCA) was performed on the correlation matrix (auto-scaled variables) using standardized formulation-level mean values of selected physical, baking, dough-texture, breadstick-texture, colour, and preliminary sensory variables. Given the limited number of formulation-level observations (n = 4), the PCA was used solely for descriptive visualization and was not interpreted as inferential multivariate evidence. The control values for crumb and crust ΔE* were set to zero by definition. Nutritional composition, final consumer-acceptance data, UHPLC-DAD-HRMS results, and antioxidant activity were excluded from the PCA. Results were presented as score and loading plots, together with the percentage of variance explained by each principal component.

## 3. Results

### 3.1. Physical and Baking Properties

The physical and baking properties of the control and fortified breadsticks are reported in Table 2. Breadstick volume and specific volume were higher in the fortified formulations than in the control, with the highest values observed for the 3.5% formulation. Baking weight loss showed relatively limited variation among formulations, whereas moisture content varied across the four products. Mean breadstick volume increased from 26.30 ± 1.20 cm^3^ in the control to 29.20 ± 1.30, 30.10 ± 1.88, and 31.60 ± 1.52 cm^3^ in the 1.5%, 2.5%, and 3.5% formulations, respectively. A similar pattern was observed for specific volume, with mean values of 2.27 ± 0.11, 2.57 ± 0.11, 2.62 ± 0.13, and 2.80 ± 0.11 cm^3^/g, respectively. Baking weight loss ranged from 29.68 ± 2.06% to 31.70 ± 1.71% across formulations. Moisture content ranged from 13.61 ± 0.53% in the control to 17.91 ± 0.44% in the 1.5% formulation, with values of 13.81 ± 0.82% and 16.86 ± 2.71% in the 2.5% and 3.5% formulations, respectively.

### 3.2. Dough and Breadstick Cutting Force

Dough-texture parameters for the four formulations are reported in Table 3. Dough hardness ranged from 19.33 ± 2.08 N in the control to 32.73 ± 1.76 N in the 2.5% formulation. The 2.5% formulation also showed the highest mean values for chewiness (1.54 ± 0.12 N) and gumminess (7.81 ± 0.22 N), whereas springiness, cohesiveness, and adhesiveness varied within narrower ranges. Maximum cutting force of the baked breadsticks ranged from 35.0 ± 9.4 N in the 1.5% formulation to 262.9 ± 24.7 N in the control, with the 2.5% and 3.5% formulations showing values of 145.6 ± 16.7 and 56.3 ± 17.2 N, respectively (Table 4).

### 3.3. Crust and Crumb Color

**Figure 1:**
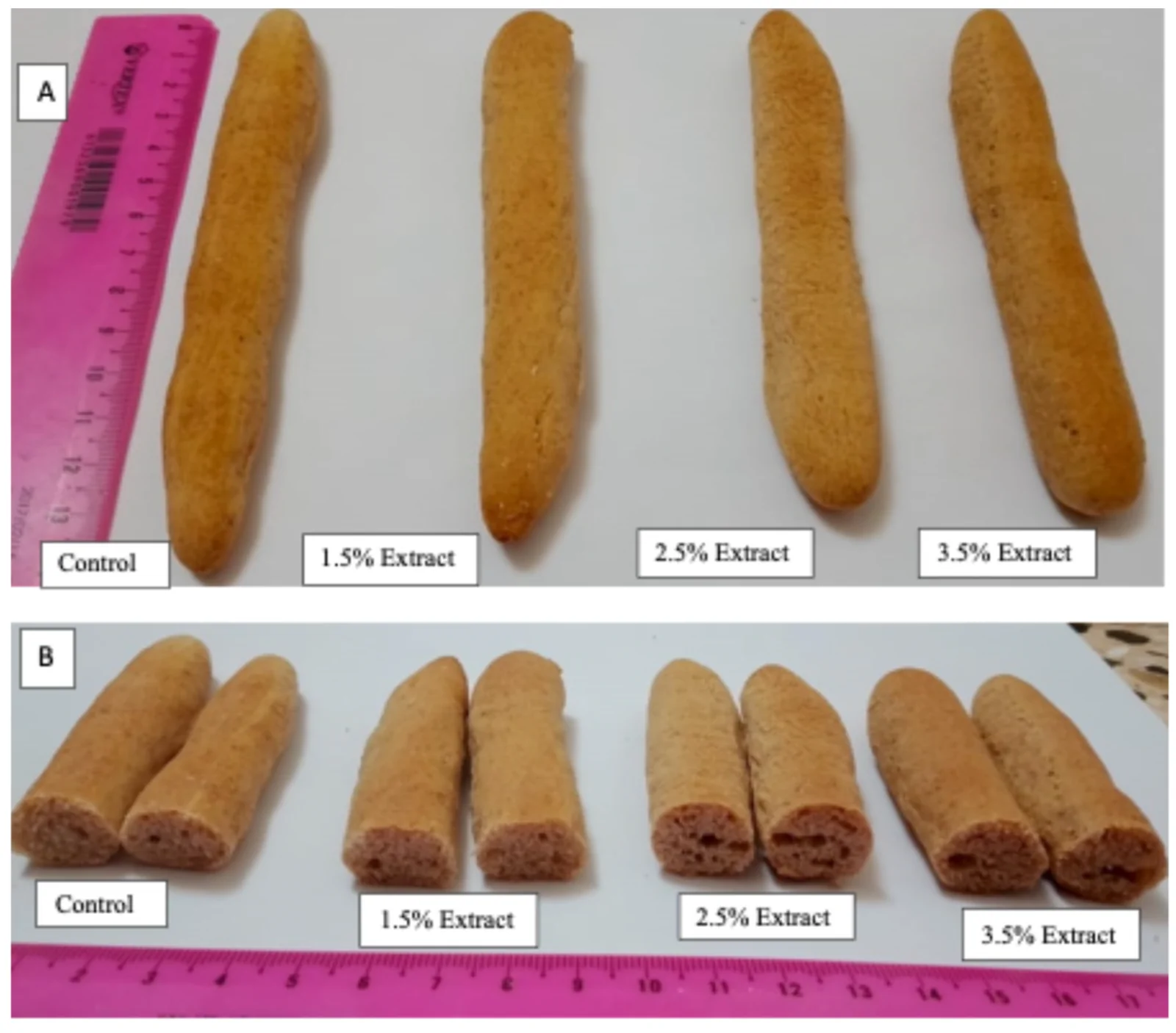
Visual appearance and internal structure of control and artichoke by-product extract-fortified breadsticks. (**A**) External appearance and (**B**) cross-sectional internal structure of the control and breadsticks containing 1.5%, 2.5%, and 3.5% artichoke by-product extract. All fortified formulations also contained 5% artichoke-derived inulin. Samples are shown from left to right in the following order: control, 1.5%, 2.5%, and 3.5% extract.

The color parameters of the crumb and crust are presented in Table 5 and Table 6. Mean crumb L* decreased from 83.42 ± 2.54 in the control to 76.40 ± 9.29, 77.26 ± 3.98, and 64.57 ± 6.37 in the 1.5%, 2.5%, and 3.5% formulations, respectively. Corresponding crumb ΔE* values were 7.15 ± 3.72, 8.32 ± 3.85, and 20.40 ± 6.85. For the crust, mean L* values were 73.39 ± 4.21 in the control and 68.36 ± 2.59, 55.40 ± 4.30, and 62.47 ± 4.42 in the fortified formulations. Crust ΔE* values were 6.73 ± 1.62, 19.32 ± 3.92, and 13.19 ± 3.28 for the 1.5%, 2.5%, and 3.5% formulations, respectively. The visual appearance of the formulations is shown in Figure 1.

### 3.4. Nutritional Composition

The nutritional composition of the breadsticks is reported in Table 7. Mean protein content ranged from 10.90 ± 0.27 to 12.30 ± 0.31 g/100 g, while lipid and ash contents showed narrow ranges across formulations. Carbohydrate content ranged from 53.31 ± 0.59 to 63.41 ± 0.62 g/100 g. Total dietary fibre increased from 4.08 ± 0.09 g/100 g in the control to 8.88 ± 0.20, 8.85 ± 0.20, and 8.81 ± 0.20 g/100 g in the 1.5%, 2.5%, and 3.5% formulations, respectively. Calculated energy values were 337, 311, 327, and 315 kcal/100 g for the control, 1.5%, 2.5%, and 3.5% formulations, respectively.

### 3.5. Preliminary Sensory Screening

A significant formulation effect was observed for colour, taste, and overall acceptability (Friedman test, *p* < 0.0001 for all attributes; Table 8). Pairwise comparisons showed no significant differences between the control and the 2.5% formulation for any of the three sensory attributes, whereas both received significantly higher ratings than the 1.5% and 3.5% formulations. Among the three fortified products, 26 of 30 participants (86.7%) selected the 2.5% formulation as their preferred sample, four participants (13.3%) selected the 1.5% formulation, and none selected the 3.5% formulation (χ^2^ = 39.20, df = 2, *p* < 0.0001).

### 3.6. Principal Component Analysis

**Figure 2:**
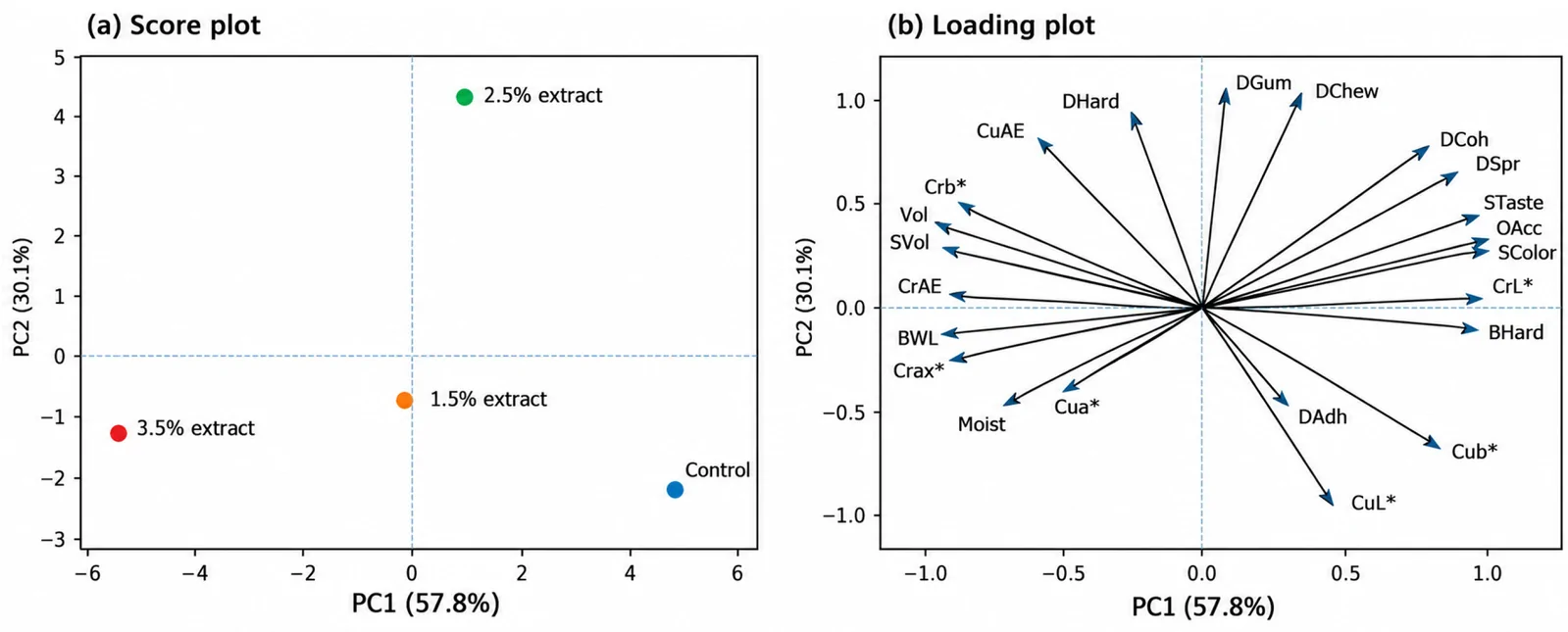
Principal component analysis (PCA) of the physical, baking, textural, colour, and preliminary sensory characteristics of control and artichoke by-product extract-fortified breadsticks. (**a**) Score plot showing the distribution of the control and formulations containing 1.5%, 2.5%, and 3.5% extract; (**b**) loading plot showing the contribution and direction of the measured variables. PC1 and PC2 explained 57.8% and 30.1% of the total variance, respectively (87.9% cumulative variance). All fortified formulations contained 5% artichoke-derived inulin. Abbreviations: Vol, volume; SVol, specific volume; BWL, baking weight loss; Moist, moisture; DHard, dough hardness; DChew, dough chewiness; DSpr, dough springiness; DCoh, dough cohesiveness; DGum, dough gumminess; DAdh, dough adhesiveness; BHard, breadstick cutting force; CrL*, Cra*, Crb*, and CrΔE*, crumb colour parameters; CuL*, Cua*, Cub*, and CuΔE*, crust colour parameters; SColor, sensory colour; STaste, sensory taste; OAcc, overall acceptability. Because the PCA was based on only four formulation-level observations, the score and loading plots should be interpreted as exploratory descriptive visualizations.

Principal component analysis was performed on the standardized formulation-level mean values of the physical, baking, dough-texture, breadstick-texture, color, and preliminary sensory variables. The first two principal components explained 87.9% of the total variance, with PC1 and PC2 accounting for 57.8% and 30.1%, respectively (Figure 2). The score plot showed spatial separation of the four formulation-level observations, while the loading plot indicated that PC1 was mainly associated with volume, specific volume, baking weight loss, crumb colour, cutting force, and sensory variables, whereas PC2 was more closely associated with dough-texture parameters and crust-colour variables. Given the limited number of formulation-level observations (n = 4), the PCA is presented only as an exploratory descriptive visualization.

### 3.7. Phytochemical Profile of the Selected Breadsticks

**Figure 3:**
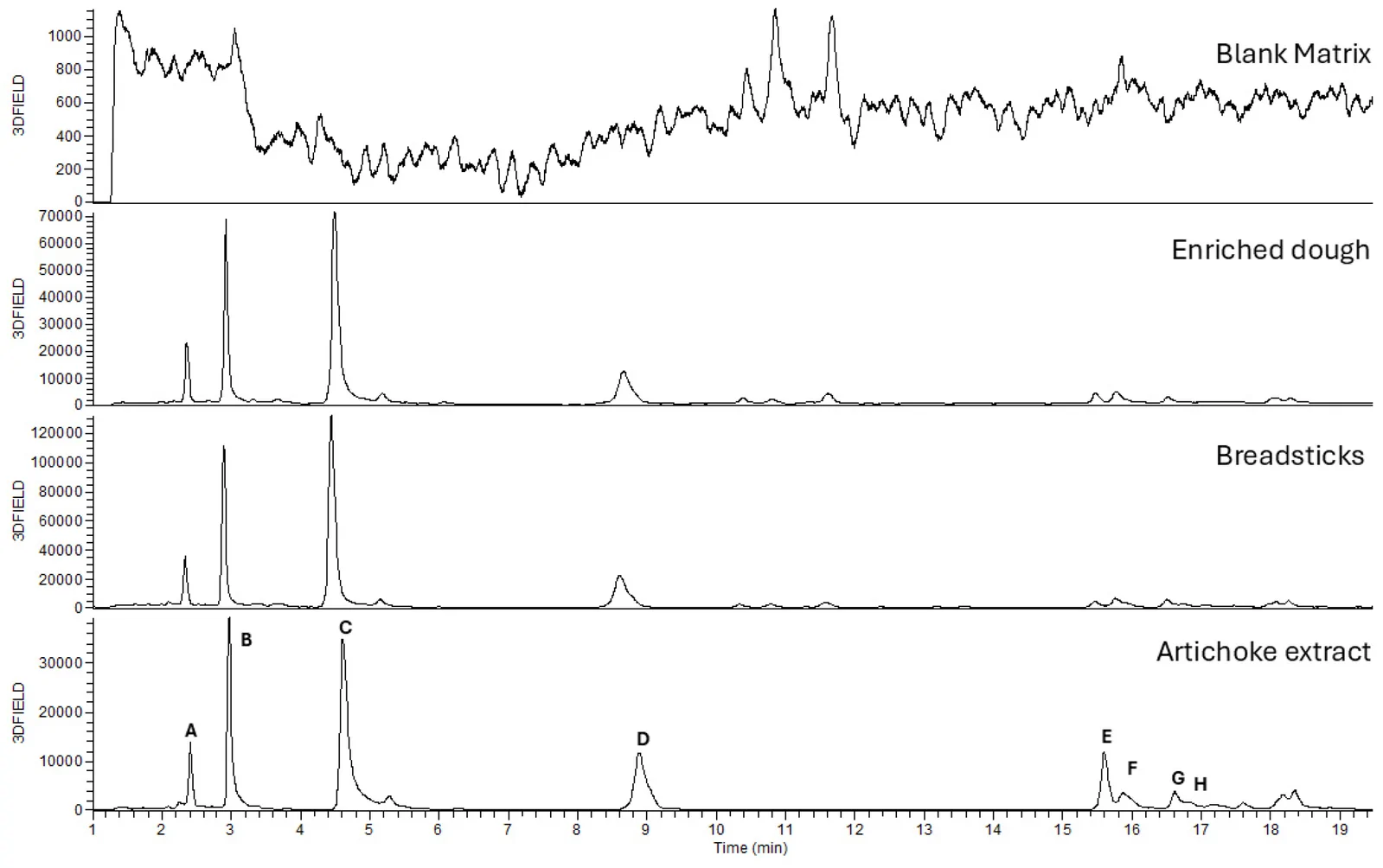
UHPLC–DAD chromatographic profiles recorded at 325 nm of the control dough, dough fortified with 2.5% artichoke by-product extract, breadsticks fortified with 2.5% artichoke extract, and the initial artichoke extract. The x-axis represents retention time (min), and the y-axis represents absorbance at 325 nm (mAU). Peak assignments (A–H) are reported in Table 9.

**Figure 4:**
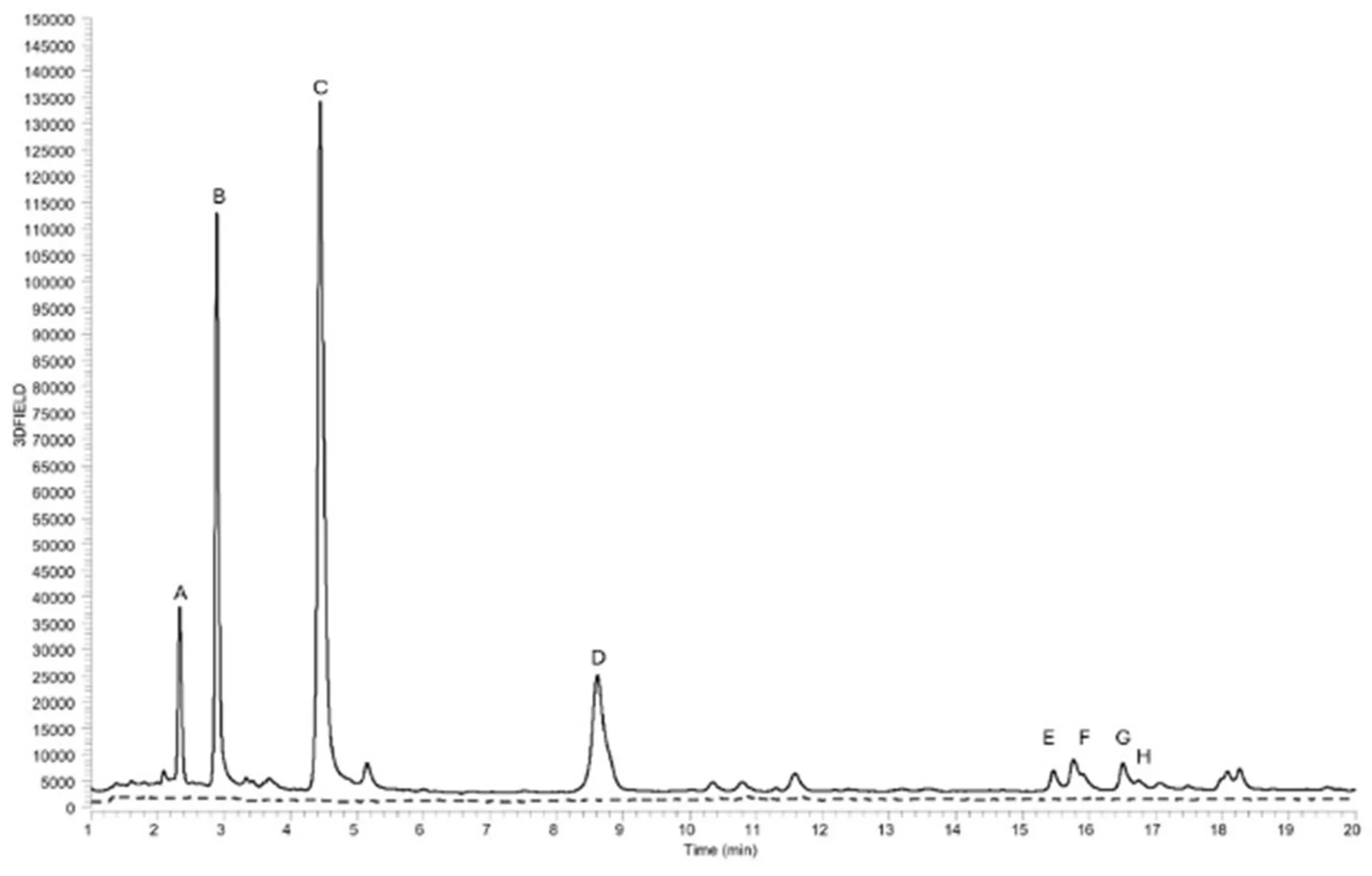
Overlaid UHPLC–DAD chromatograms recorded at 325 nm of the control breadsticks and breadsticks fortified with 2.5% artichoke by-product extract. The *x*-axis represents retention time (min), and the *y*-axis represents absorbance at 325 nm (mAU). Control and fortified samples are represented by dashed and solid lines, respectively. Peak assignments (A–H) are reported in Table 9.

UHPLC–DAD–HRMS/MS analysis was performed to characterize the phytochemical profile of the artichoke by-product extract and to verify the presence of its principal phenolic constituents in the selected formulation before and after baking. The chromatographic profiles recorded at 325 nm for the control dough, dough fortified with 2.5% artichoke extract, fortified breadsticks, and the initial artichoke extract are shown in Figure 3. The fortified dough exhibited a chromatographic pattern consistent with that of the initial extract, with signals corresponding to the major caffeoylquinic acid derivatives. These characteristic signals remained detectable in the fortified breadsticks after baking, although differences in peak intensity were observed between the dough and the final product. The comparison between the control and fortified breadsticks further confirmed that the principal signals observed in the selected formulation were associated with the incorporated artichoke extract (Figure 4). The control breadsticks showed substantially lower responses at the retention times of the main artichoke-derived constituents, whereas the fortified breadsticks displayed the characteristic chromatographic profile of mono- and dicaffeoylquinic acids and luteolin derivatives.

Eight phenolic compounds were identified or putatively annotated by combining retention time, accurate mass, molecular formula, MS/MS fragmentation, UV spectral data, and comparison with authentic standards where available (Table 9). Three mono-caffeoylquinic acid derivatives were detected at retention times of 2.4, 3.0, and 4.5 min, all showing a deprotonated molecular ion at *m*/*z* 353.0876 and characteristic fragment ions at *m*/*z* 191, 179, and 173. The compound eluting at 4.5 min was confirmed as chlorogenic acid by comparison with an authentic standard, whereas the other two compounds were putatively annotated as caffeoylquinic acid isomers. Cynarin was identified at 8.6 min based on its deprotonated molecular ion at *m*/*z* 515.1194, fragment ions at *m*/*z* 353, 191, and 179, and comparison with an authentic standard. Two additional dicaffeoylquinic acid derivatives displaying the same precursor and product ions were detected at 16.5 and 16.8 min and were putatively annotated as dicaffeoylquinic acid isomers. Luteolin-7-*O*-glucoside was confirmed at 15.5 min by comparison with an authentic standard, while luteolin *O*-glucuronide was putatively annotated at 15.8 min based on its deprotonated molecular ion at *m*/*z* 461.0726 and the characteristic aglycone fragment at *m*/*z* 285. The eight annotated compounds were subsequently included in the quantitative analysis reported in Section 3.8.

### 3.8. Quantitative Phenolic Composition, Baking Retention, and Antioxidant Activity

Quantitative UHPLC–DAD analysis was performed on the initial artichoke by-product extract, the fortified dough before baking, and the corresponding baked breadsticks containing 2.5% artichoke extract and 5% artichoke-derived inulin (Table 10). Chlorogenic acid was the predominant compound in the initial extract, reaching 48.44 ± 0.39 mg/g dry extract, in agreement with the nominal standardization of the extract to 5% chlorogenic acid. The sum of caffeoylquinic acid derivatives in the initial extract was 59.39 ± 1.04 mg/g, while luteolin derivatives accounted for 4.08 ± 0.16 mg/g.

In the fortified dough, the sum of caffeoylquinic acid derivatives was 67.27 ± 1.03 mg/100 g, whereas luteolin derivatives accounted for 3.76 ± 0.23 mg/100 g. After baking, the corresponding concentrations were 79.17 ± 2.07 and 4.13 ± 0.09 mg/100 g, respectively. The total concentration of the eight quantified phenolic compounds in the baked breadsticks was 83.30 ± 1.99 mg/100 g on an as-is basis. Chlorogenic acid remained the most abundant individual compound in the final product at 35.75 ± 1.01 mg/100 g, followed by the caffeoylquinic acid isomer B at 17.17 ± 0.59 mg/100 g and cynarin at 14.56 ± 1.23 mg/100 g.

Because baking caused a mean mass loss of 30.48% in the selected formulation, phenolic retention was calculated on a mass basis rather than from the apparent concentration change alone. After correction for baking mass loss, the overall retention of the total quantified phenolic fraction was 81.5%. Caffeoylquinic acid derivatives showed an overall baking retention of 81.8%, with individual values of 83.2% for chlorogenic acid and 78.2% for cynarin. The summed luteolin derivatives showed a retention of 76.3%. Individual retention values ranged from 59.6% for luteolin-7-*O*-glucoside to 91.9% for luteolin-7-*O*-glucuronide. After correction for baking mass loss, total quantified phenolic retention was 81.5%, caffeoylquinic acid retention was 81.8%, and summed luteolin retention was 76.3. The selected breadsticks showed an ABTS radical-scavenging capacity of 35.65 ± 2.05 µmol TE/g, corresponding to 3.57 ± 0.21 mmol TE/100 g.

### 3.9. Fructan Content

The fructan content of the selected 2.5% formulation was 4.92 ± 0.21 g/100 g of breadsticks, based on three analytical determinations. The relative standard deviation was <5%.

### 3.10. Consumer Acceptance of the Selected Formulation

**Figure 5:**
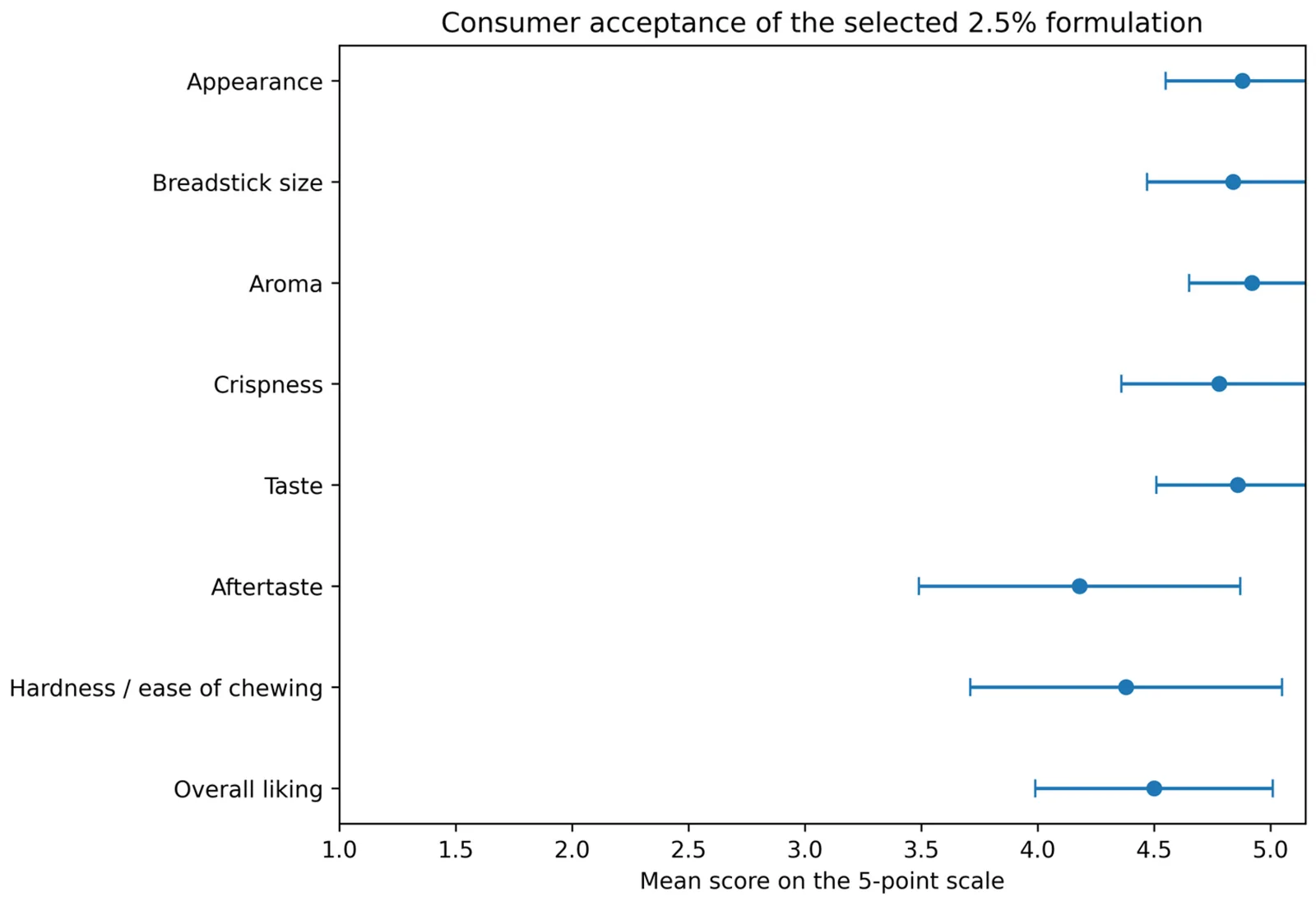
Consumer acceptance of breadsticks containing 2.5% artichoke by-product extract and 5% artichoke-derived inulin. Mean sensory scores ± standard deviation obtained from 50 consumers using a 5-point acceptance scale.

Mean scores ranged from 4.18 ± 0.69 to 4.92 ± 0.27 on the 5-point scale (Table 11). Aroma showed the highest mean score (4.92 ± 0.27), followed by appearance (4.88 ± 0.33), taste (4.86 ± 0.35), breadstick size (4.84 ± 0.37), and crispness (4.78 ± 0.42). Overall liking was 4.50 ± 0.51, with all 50 consumers assigning a score of either 4 or 5. Lower, although still favorable, ratings were obtained for ease of chewing (4.38 ± 0.67) and aftertaste (4.18 ± 0.69). Positive ratings (scores 4–5) were recorded for 90% of responses for ease of chewing and 84% for aftertaste. No score below 3 was recorded for any attribute. The mean consumer scores and their variability are graphically summarized in Figure 5.

## 4. Discussion

The present study demonstrated that a standardized artichoke by-product extract and purified artichoke-derived inulin can be jointly incorporated into wheat breadsticks to improve their fibre and phytochemical profiles while maintaining acceptable technological and sensory characteristics. This approach differs from most fortification studies, which generally replace part of the wheat flour with relatively high amounts of whole plant or by-product powders [1,2,21]. The use of two separately recovered fractions allowed the fibre contribution to be provided mainly by inulin, while the extract supplied characteristic artichoke phenolics at lower inclusion levels. However, because the control contained neither ingredient, differences between the control and fortified products reflect their combined effects, whereas comparisons among the three fortified formulations primarily reflect the increasing extract concentration.

The response of breadstick expansion differed from that commonly reported after incorporation of fibre-rich plant powders. Mean volume increased from 26.30 cm^3^ in the control to 29.20, 30.10, and 31.60 cm^3^ in the 1.5%, 2.5%, and 3.5% formulations, respectively, while specific volume increased from 2.27 to 2.57, 2.62, and 2.80 cm^3^/g. By contrast, Rainero et al. reported decreases in specific volume from 3.82 cm^3^/g in control breadsticks to 2.43 and 1.82 cm^3^/g after incorporation of 5% and 10% grape pomace powder [3].

This opposite response may reflect the different physical nature of the incorporated ingredients. Whole grape pomace introduces insoluble particulate material that may interfere with gluten development, whereas the present formulation combined a soluble phenolic extract with purified inulin [1,2,22]. Inulin may also modify water distribution, dough viscosity, and gas retention during fermentation and baking [23]. Baking weight loss varied only within a narrow range (29.68–31.70%), but because water addition differed among formulations and no inulin-only formulation was included, the greater expansion cannot be attributed specifically to either the phenolic extract or inulin.

A distinctive response emerged for texture. In the 2.5% formulation, dough hardness increased from 19.33 to 32.73 N (+69%), gumminess from 4.31 to 7.81 N (+81%), and chewiness from 0.82 to 1.54 N (+88%) relative to the control, whereas maximum cutting force of the baked breadsticks decreased from 262.9 to 145.6 N (−45%). Thus, greater resistance to deformation in the fermented dough did not translate into a harder baked structure. This may reflect the combined effects of starch gelatinization, gluten restructuring, moisture redistribution, and inulin-mediated changes in water availability and starch–gluten interactions [22,23]. Soluble fibres such as inulin can also modify wheat-dough rheology and gluten-network organization through water interactions and hydrogen bonding [24].

The reduction in baked-product resistance is consistent with the direction reported for grape-pomace-enriched breadsticks [3], whereas *Wolffia globosa*, pearl millet/whey protein, and *Ziziphus spina-christi* fortification produced increasing hardness at higher substitution levels [4,25,26]. These contrasting responses indicate that mechanical behaviour depends not only on fibre level but also on the physical nature of the added ingredient. The intermediate cutting force of the 2.5% product may therefore represent a useful structural compromise.

Marked colour changes were observed in the fortified breadsticks, although the response was not concentration-dependent. In the crumb, L* decreased from 83.42 in the control to 76.40, 77.26, and 64.57 in the 1.5%, 2.5%, and 3.5% formulations, respectively, while ΔE* increased from 7.15 and 8.32 at the two lower extract levels to 20.40 at 3.5%. A different pattern was observed for the crust, where the greatest colour difference occurred in the 2.5% formulation (ΔE* = 19.32), compared with 6.73 at 1.5% and 13.19 at 3.5%.

Comparable darkening has been reported for breadsticks fortified with other plant-derived ingredients. Rainero et al. observed a decrease in L* from 92.22 in control breadsticks to 69.98 and 65.02 after incorporation of 5% and 10% grape pomace powder, with corresponding ΔE* values of 23.59 and 29.36 [3]. The colour changes observed in the present study were therefore of a similar direction but, at moderate extract incorporation, generally of lower magnitude than those reported for whole grape-pomace powder. This difference may reflect the use of a fractionated phenolic extract rather than a highly pigmented whole plant matrix.

The non-monotonic crust response also suggests an important contribution of processing variables. Phenolic compounds may directly affect product colour and undergo oxidation or thermal transformation during baking, while differences in initial hydration and final moisture can modify heat transfer, water evaporation, and browning reactions [1,23]. Because water addition differed among formulations, the observed colour changes cannot be attributed exclusively to the amount of artichoke extract. Importantly, the large instrumental colour difference of the 2.5% formulation did not translate into reduced sensory acceptance relative to the control, whereas the 3.5% formulation, which showed the greatest crumb colour change, also received the lowest taste and overall-acceptability scores. Thus, the magnitude of instrumental colour change was not directly mirrored by sensory acceptance.

Dietary fibre enrichment was one of the most pronounced nutritional effects of fortification. Total dietary fibre increased from 4.08 g/100 g in the control to 8.88, 8.85, and 8.81 g/100 g in the fortified formulations, while the selected 2.5% formulation contained 4.92 ± 0.21 g/100 g of fructans. The narrow range among fortified samples indicates that the increase was driven primarily by the fixed 5% addition of purified artichoke-derived inulin, consistent with the abundance of inulin-type fructans in artichoke stems and bracts [9,10].

Cannas et al. reported increases in total dietary fibre from 5.00 g/100 g dry matter in control breadsticks to 7.59 and 6.52 g/100 g after incorporation of 5% outer-bract and stem powders, respectively [11]. Although these values were expressed on a dry-matter basis and cannot be directly equated with the present as-is data, the comparison highlights the different impact of the two strategies. In the present study, purified inulin produced an approximately twofold increase in fibre content without introducing the whole structural plant matrix, whereas whole powders simultaneously introduce fibre, phenolics, pigments, volatile compounds, and insoluble structural components that may exert broader effects on dough and sensory properties.

This distinction is further illustrated by previous artichoke-based bakery studies. Cannas et al. showed that polyphenol-rich hydroalcoholic extracts from artichoke bracts, leaves, and stems could increase antioxidant capacity and oxidative stability in breadsticks with relatively limited effects on dough workability [21], while Bavaro et al. identified 10% artichoke bract powder as a suitable technological and sensory compromise in bread and reported chlorogenic and dicaffeoylquinic acids among the principal bioaccessible phenolic compounds [27]. However, these approaches either introduced the whole by-product matrix or focused on the phenolic fraction as a single fortifying ingredient. The present strategy combined two separately recovered fractions from the same artichoke biomass: purified inulin at a fixed concentration and a standardized phenolic extract at increasing levels. This design allowed fibre enrichment and phenolic fortification to be modulated more independently, while also enabling quantitative tracking of characteristic phenolic markers through dough processing and baking. The novelty of the present work therefore lies not simply in the use of artichoke by-products, but in the integration of a fractionation-based formulation strategy with mass-corrected assessment of phenolic retention during processing.

UHPLC-DAD-HRMS/MS showed that caffeoylquinic acid derivatives were the predominant phenolic class in the selected breadsticks, with chlorogenic acid and cynarin among the major individual compounds. In the baked product, the total concentration of the eight quantified phenolic markers was 83.30 mg/100 g, of which 79.17 mg/100 g corresponded to caffeoylquinic acid derivatives. Because baking caused a 30.48% mass loss, retention was evaluated on a mass-corrected basis rather than from concentration changes alone. Overall retention of the quantified phenolic fraction was 81.5%, with 81.8% retention for caffeoylquinic acid derivatives and 76.3% for luteolin derivatives. These values indicate that the principal artichoke-derived phenolic markers were largely preserved during baking, while also showing clear compound-dependent behaviour.

The individual retention values provide further insight into processing-related transformations. Chlorogenic acid and cynarin showed retentions of 83.2% and 78.2%, respectively, whereas the two luteolin derivatives behaved differently, with 59.6% retention for luteolin-7-*O*-glucoside and 91.9% for luteolin-7-*O*-glucuronide. For caffeoylquinic acids, changes in individual marker abundance may reflect not only thermal degradation but also heat-induced acyl migration and isomerization among 3-O-, 4-O-, and 5-O-caffeoylquinic acid forms, as previously described [28,29]. Likewise, differences in luteolin recovery may reflect differences in thermal stability and matrix interactions that affect extractability after baking [30]. Because several compounds were quantified using representative class standards and thermal interconversion among related phenolics cannot be resolved from the present dataset, class-level retention provides a more robust indicator of overall process stability than the behaviour of individual markers.

The selected breadsticks showed an ABTS radical-scavenging capacity of 35.65 ± 2.05 µmol TE/g, corresponding to 3.57 ± 0.21 mmol TE/100 g. This value demonstrates measurable in vitro radical-scavenging capacity in the final baked product and is consistent with previous studies reporting enhanced DPPH and ABTS responses after incorporation of artichoke-derived extracts into breadsticks [21]. However, direct quantitative comparison across studies is limited by differences in ingredient type, fortification level, food matrix, analytical expression, and assay conditions. Moreover, the measured ABTS response cannot be attributed exclusively to the quantified artichoke phenolics, because cereal constituents, unquantified phytochemicals, and compounds formed during baking may also contribute. Accordingly, the ABTS value should be interpreted as an indicator of in vitro radical-scavenging potential rather than as evidence of physiological antioxidant efficacy.

The biological relevance of retained phenolics also depends on their release during gastrointestinal digestion. In artichoke stem-enriched bread, Colantuono et al. reported 82% bioaccessibility of total polyphenols and 74% bioaccessibility of cynaropicrin, together with measurable antioxidant and α-glucosidase inhibitory activity [31]. Vacca et al. likewise reported retained antioxidant and anti-inflammatory activity after in vitro digestion of gluten-free bread enriched with artichoke leaf extract [32]. These findings support the potential gastrointestinal availability of artichoke-derived phenolics, but they cannot be directly extrapolated to the present formulation. Since bioaccessibility was not assessed here, the present results support phenolic retention and in vitro antioxidant potential, but not gastrointestinal or in vivo efficacy.

Sensory response showed a clear formulation-dependent pattern, with the 2.5% extract level providing the most favourable balance among the fortified products. Its colour, taste, and overall acceptability scores did not differ significantly from the control, whereas the 3.5% formulation showed substantially lower ratings. Consistently, 26 of 30 participants (86.7%) selected the 2.5% formulation as their preferred fortified product, while none selected the 3.5% formulation. Similar concentration-dependent effects have been reported for Wolffia globosa- and Ziziphus spina-christi-fortified bakery products [4,26]. In artichoke-fortified products, increasing levels of phenolic and volatile compounds may also contribute bitter, astringent, and herbaceous notes [11], although these attributes were not measured directly in the present study.

The selected 2.5% formulation also maintained favourable consumer acceptance, with crispness and taste scores of 4.78 ± 0.42 and 4.86 ± 0.35 and overall liking of 4.50 ± 0.51. Aftertaste remained the lowest-rated attribute (4.18 ± 0.69), although 84% of responses were positive. The PCA provided a complementary descriptive visualization, but given the four formulation-level observations, it should be interpreted solely as exploratory support rather than confirmatory evidence.

Several limitations should nevertheless be considered when interpreting these findings. The experimental design did not include formulations containing inulin alone or extract alone, and water addition differed among recipes; therefore, the individual contribution of each ingredient to dough and breadstick properties cannot be fully resolved. Quantitative phenolic tracking was performed only for the selected 2.5% formulation, and the calculated retention values refer to the quantified marker compounds rather than to the complete phenolic fraction. In addition, gastrointestinal bioaccessibility, shelf-life, oxidative stability, and glycaemic response were not assessed, and the final consumer test did not include a simultaneous control product. Furthermore, only one production and baking batch was prepared for each formulation; therefore, the technical and analytical replicates characterize within-batch variability but do not establish between-batch reproducibility.

These limitations are consistent with broader gaps identified in the literature on fortified bakery products, including limited sensory sample sizes, scarce assessment of phytochemical bioavailability, and insufficient information on shelf-life and long-term functional performance [1]. Nevertheless, the selected formulation combined several practically relevant features: 8.85 g/100 g total dietary fibre, 4.92 g/100 g fructans, 81.5% retention of the quantified phenolic markers after baking, and an overall consumer-liking score of 4.50 ± 0.51/5. From an application perspective, these results support the feasibility of using separately recovered artichoke-derived inulin and phenolic fractions as complementary ingredients in bakery formulations, allowing fibre enrichment and phytochemical fortification to be adjusted more independently than with whole by-product powders.

## 5. Conclusions

The combined use of a standardized artichoke by-product extract and purified artichoke-derived inulin enabled the development of high-fibre wheat breadsticks with satisfactory technological and sensory characteristics. Among the tested formulations, the combination of 2.5% artichoke extract and 5% inulin provided the most balanced profile, maintaining sensory acceptability comparable to the control while increasing total dietary fibre to 8.85 g/100 g and providing 4.92 ± 0.21 g/100 g of fructans. Targeted UHPLC-DAD-HRMS/MS analysis confirmed the presence of characteristic artichoke-derived caffeoylquinic acids and luteolin derivatives throughout processing. The selected baked breadsticks contained 83.30 ± 1.99 mg/100 g of quantified phenolic compounds. Mass-balance correction for baking weight loss showed an overall retention of 81.5% for the eight quantified phenolic markers, with 81.8% retention of caffeoylquinic acid derivatives. The final product also exhibited measurable in vitro antioxidant capacity and favorable consumer liking, with an overall liking score of 4.50 ± 0.51. These findings demonstrate the potential of separately recovered phenolic and inulin fractions from artichoke processing residues as complementary ingredients for the development of nutritionally enhanced bakery products and support an integrated strategy for agro-industrial by-product valorization. Further studies addressing gastrointestinal bioaccessibility, shelf-life, and validation in larger consumer populations would help define the nutritional and functional relevance of the developed product.

## Figures and Tables

**Table 1:** Calibration parameters for the quantitative determination of selected phenolic compounds by UHPLC-DAD-HRMS/MS.

Compound	Linear Range (µg mL^−1^)	Calibration Equation	R^2^	LOD (mg g^−1^)	LOQ (mg g^−1^)
Chlorogenic acid	1–150	y = 0.6402x − 1.0635	0.9997	0.58	1.76
Cynarin	1–150	y = 0.7498x − 0.3919	0.9998	0.61	1.85
Luteolin-7-*O*-glucoside	1–150	y = 0.8540x − 0.6970	0.9995	0.66	1.98

LOD, limit of detection; LOQ, limit of quantification.

**Table 2:** Physical and baking properties of control and artichoke by-product extract- and inulin-fortified breadsticks.

Formulation	Volume (cm^3^)	Specific Volume (cm^3^ g^−1^)	Baking Weight Loss (%)	Moisture Content (%)
Control	26.30 ± 1.20	2.27 ± 0.11	29.68 ± 2.06	13.61 ± 0.53
1.5% extract	29.20 ± 1.30	2.57 ± 0.11	31.07 ± 0.57	17.91 ± 0.44
2.5% extract	30.10 ± 1.88	2.62 ± 0.13	30.48 ± 1.79	13.81 ± 0.82
3.5% extract	31.60 ± 1.52	2.80 ± 0.11	31.70 ± 1.71	16.86 ± 2.71

Values are expressed as mean ± standard deviation of three technical measurements performed on samples obtained from a single baking batch for each formulation. Data are presented descriptively; no inferential statistical comparisons were performed.

**Table 3:** Textural properties of control and artichoke by-product extract- and inulin-fortified breadstick doughs.

Formulation	Hardness (N)	Chewiness (N)	Springiness	Cohesiveness	Gumminess (N)	Adhesiveness Force (N)
Control	19.33 ± 2.08	0.82 ± 0.33	0.19 ± 0.03	0.220 ± 0.040	4.31 ± 1.29	−1.070 ± 0.030
1.5% extract	23.23 ± 2.73	0.87 ± 0.13	0.18 ± 0.01	0.210 ± 0.003	4.93 ± 0.60	−1.100 ± 0.010
2.5% extract	32.73 ± 1.76	1.54 ± 0.12	0.20 ± 0.01	0.240 ± 0.010	7.81 ± 0.22	−1.090 ± 0.008
3.5% extract	24.46 ± 0.97	0.72 ± 0.17	0.16 ± 0.02	0.190 ± 0.030	4.62 ± 0.61	−1.080 ± 0.006

Values are expressed as mean ± standard deviation of five technical measurements performed on dough obtained from a single production batch for each formulation. Data are presented descriptively; no inferential statistical comparisons were performed.

**Table 4:** Maximum cutting force of control and artichoke by-product extract- and inulin-fortified breadsticks.

Formulation	Maximum Cutting Force (N)
Control	262.9 ± 24.7
1.5% extract	35.0 ± 9.4
2.5% extract	145.6 ± 16.7
3.5% extract	56.3 ± 17.2

Values are expressed as mean ± standard deviation of five breadsticks obtained from a single baking batch for each formulation. Data are presented descriptively; no inferential statistical comparisons were performed.

**Table 5:** Crumb color parameters of control and artichoke by-product extract- and inulin-fortified breadsticks.

Formulation	L*	a*	b*	ΔE*
Control	83.42 ± 2.54	11.74 ± 1.06	17.80 ± 5.82	—
1.5% extract	76.40 ± 9.29	15.11 ± 4.70	18.71 ± 7.14	7.15 ± 3.72
2.5% extract	77.26 ± 3.98	13.53 ± 2.56	19.90 ± 4.31	8.32 ± 3.85
3.5% extract	64.57 ± 6.37	18.26 ± 2.33	20.79 ± 3.33	20.40 ± 6.85

Values are expressed as mean ± standard deviation of five technical measurements performed on breadsticks obtained from a single production batch for each formulation. Data are presented descriptively; no inferential statistical comparisons were performed. L, a*, b* and ΔE* are defined as described in Section 2.14.

**Table 6:** Crust colour parameters of control and artichoke by-product extract- and inulin-fortified breadsticks.

Formulation	L*	a*	b*	ΔE*
Control	73.39 ± 4.21	23.83 ± 1.12	36.86 ± 4.55	—
1.5% extract	68.36 ± 2.59	21.32 ± 1.07	34.57 ± 2.05	6.73 ± 1.62
2.5% extract	55.40 ± 4.30	23.33 ± 3.68	31.43 ± 2.33	19.32 ± 3.92
3.5% extract	62.47 ± 4.42	25.15 ± 1.89	30.89 ± 2.64	13.19 ± 3.28

Values are expressed as mean ± standard deviation of five technical measurements performed on breadsticks obtained from a single production batch for each formulation. Data are presented descriptively; no inferential statistical comparisons were performed. *L*, *a*, b* and ΔE* are defined as described in Section 2.14.

**Table 7:** Nutritional composition and energy value of control and artichoke by-product extract- and inulin-fortified breadsticks.

Nutritional Trait	Control	1.5% Extract	2.5% Extract	3.5% Extract
Moisture (g/100 g)	13.61 ± 0.53	17.91 ± 0.44	13.81 ± 0.82	16.86 ± 2.71
Protein (g/100 g)	10.90 ± 0.27	11.80 ± 0.30	12.10 ± 0.30	12.30 ± 0.31
Lipids (g/100 g)	3.50 ± 0.09	3.60 ± 0.09	3.60 ± 0.09	3.60 ± 0.09
Carbohydrates (g/100 g)	63.41 ± 0.62	53.31 ± 0.59	57.14 ± 0.91	53.93 ± 2.74
Total dietary fibre (g/100 g)	4.08 ± 0.09	8.88 ± 0.20	8.85 ± 0.20	8.81 ± 0.20
Ash (g/100 g)	4.47 ± 0.11	4.53 ± 0.11	4.49 ± 0.11	4.51 ± 0.11
Energy (kcal/100 g)	337	311	327	315
Energy (kJ/100 g)	1410	1300	1368	1318

Values are expressed per 100 g of breadsticks on an as-is basis. Moisture, protein, lipid, total dietary fibre, and ash values are reported as mean ± standard deviation of three analytical determinations performed on samples obtained from a single baking batch per formulation. Carbohydrates were calculated by difference for each analytical determination and are reported as mean ± standard deviation. Energy values were calculated from the mean compositional data and are reported without standard deviations. Data are presented descriptively; no inferential statistical comparisons among formulations were performed.

**Table 8:** Preliminary sensory evaluation and preference of control and artichoke by-product extract- and inulin-fortified breadsticks.

Formulation	Color	Taste	Overall Acceptability	Preferred Enriched Formulation, n (%)
Control	8.53 ± 0.68^a^	8.40 ± 0.62^a^	8.43 ± 0.63^a^	—
1.5% extract	7.50 ± 1.07^b^	7.13 ± 1.17^b^	7.33 ± 1.09^b^	4 (13.3%)
2.5% extract	8.37 ± 1.07^a^	8.36 ± 0.80^a^	8.30 ± 0.70^a^	26 (86.7%)
3.5% extract	5.17 ± 0.83^c^	4.33 ± 1.37^c^	4.23 ± 1.01^c^	0 (0.0%)
*p*-value	<0.0001	<0.0001	<0.0001	<0.0001

Sensory scores are expressed as mean ± standard deviation for 30 participants using a 9-point hedonic scale. Different lowercase superscript letters within the same column indicate significant differences among formulations according to the Friedman test followed by pairwise Wilcoxon signed-rank tests with Holm adjustment (*p* < 0.05). Preference frequencies refer exclusively to the three enriched formulations and were evaluated using a chi-square goodness-of-fit test against an equal expected distribution.

**Table 9:** UHPLC-DAD-HRMS/MS data of phenolic compounds detected in the artichoke by-product extract and identified in the selected fortified breadsticks.

Index	Compound	RT (min)	Molecular Formula	[M−H]− (*m*/*z*)	Product Ions (*m*/*z*)	MSI Level
A	Caffeoylquinic acid	2.4	C_16_H_18_O_9_	353.0876	191, 179, 173	2
B	Caffeoylquinic acid isomer	3.0	C_16_H_18_O_9_	353.0876	191, 179, 173	2
C	Chlorogenic acid	4.5	C_16_H_18_O_9_	353.0876	191, 179, 173	1
D	Cynarin	8.6	C_25_H_24_O_12_	515.1194	353, 191, 179	1
E	Luteolin-7-*O*-glucoside	15.5	C_21_H_20_O_11_	447.0933	285	1
F	Luteolin-7-*O*-glucuronide	15.8	C_21_H_18_O_12_	461.0726	285	2
G	Dicaffeoylquinic acid	16.5	C_25_H_24_O_12_	515.1194	353, 191, 179	2
H	Dicaffeoylquinic acid isomer	16.8	C_25_H_24_O_12_	515.1194	353, 191, 179	2

RT, retention time. MSI Level 1 indicates identification confirmed by comparison with an authentic reference standard; MSI Level 2 indicates putative annotation based on accurate mass, MS/MS fragmentation, UV data, and comparison with literature or spectral databases.

**Table 10:** Quantitative phenolic composition of the initial artichoke by-product extract, fortified dough, and baked breadsticks, with mass-corrected baking retention for the selected 2.5% formulation.

Compound	Initial Extract (mg/g)	Fortified Dough (mg/100 g)	Baked Breadsticks (mg/100 g)	Baking Retention (%)
A–Caffeoylquinic acid	1.03 ± 0.04	5.07 ± 0.12	6.26 ± 0.31	85.9
B–Caffeoylquinic acid isomer	3.99 ± 0.15	13.78 ± 0.51	17.17 ± 0.59	86.6
C–Chlorogenic acid	48.44 ± 0.39	29.85 ± 0.03	35.75 ± 1.01	83.2
D–Cynarin	4.26 ± 0.23	12.95 ± 0.40	14.56 ± 1.23	78.2
E–Luteolin-7-*O*-glucoside	2.51 ± 0.13	1.81 ± 0.03	1.55 ± 0.01	59.6
F–Luteolin-7-*O*-glucuronide	1.57 ± 0.02	1.95 ± 0.19	2.58 ± 0.09	91.9
G–Dicaffeoylquinic acid	0.88 ± 0.12	2.71 ± 0.14	2.70 ± 0.14	69.3
H–Dicaffeoylquinic acid isomer	0.79 ± 0.11	2.91 ± 0.42	2.73 ± 0.30	65.3
Sum caffeoylquinic acid derivatives	59.39 ± 1.04	67.27 ± 1.03	79.17 ± 2.07	81.8
Sum luteolin derivatives	4.08 ± 0.16	3.76 ± 0.23	4.13 ± 0.09	76.3
Total quantified phenolics	63.48 ± 1.20	71.03 ± 1.26	83.30 ± 1.99	81.5

Values are expressed as mean ± standard deviation of three analytical determinations. Concentrations in the initial artichoke by-product extract are expressed as mg g^−1^ dry extract, whereas concentrations in the fortified dough and baked breadsticks are expressed as mg/100 g on an as-is basis. Mono-caffeoylquinic acid derivatives (A–C) were quantified as chlorogenic acid equivalents; cynarin and the other dicaffeoylquinic acid derivatives (D, G, and H) as cynarin equivalents; and luteolin derivatives (E and F) as luteolin-7-*O*-glucoside equivalents. Baking retention was calculated on a mass basis after correction for the experimentally determined baking weight loss of the selected 2.5% formulation (30.48%).

**Table 11:** Descriptive statistics and score distribution for the sensory attributes of breadsticks containing 2.5% artichoke by-product extract and 5% artichoke-derived inulin (n = 50).

**Sensory Attribute**	**Mean ± SD**	**Median (IQR)**	**Score 3, n (%)**	**Score 4, n (%)**	**Score 5, n (%)**	**Ratings 4–5 (%)**
Appearance	4.88 ± 0.33	5 (0)	0 (0)	6 (12)	44 (88)	100
Breadstick size	4.84 ± 0.37	5 (0)	0 (0)	8 (16)	42 (84)	100
Aroma	4.92 ± 0.27	5 (0)	0 (0)	4 (8)	46 (92)	100
Crispness	4.78 ± 0.42	5 (0)	0 (0)	11 (22)	39 (78)	100
Taste	4.86 ± 0.35	5 (0)	0 (0)	7 (14)	43 (86)	100
Aftertaste	4.18 ± 0.69	4 (1)	8 (16)	25 (50)	17 (34)	84
Ease of chewing	4.38 ± 0.67	4 (1)	5 (10)	21 (42)	24 (48)	90
Overall liking	4.50 ± 0.51	4.5 (1)	0 (0)	25 (50)	25 (50)	100

Consumer responses were recorded using a 5-point scale, where higher values indicated greater acceptance. IQR, interquartile range. Ratings of 4 or 5 were considered positive responses. Consumer data were analyzed descriptively.

## Data Availability

The original contributions presented in this study are included in the article/Supplementary Materials. Further inquiries can be directed to the corresponding author.

## Supplementary Materials

The following supporting information can be downloaded at: https://www.mdpi.com/article/10.3390/foods15213884/s1, Questionnaire S1: Preliminary sensory evaluation questionnaire; Questionnaire S2: Consumer acceptance questionnaire for the selected breadstick formulation.
