# Supplementary material for: High-Fibre Breadsticks Fortified with Artichoke By-Product Extract and Inulin: Technological Quality, Phenolic Retention, and Consumer Acceptance
Source: Foods. 2026 Oct 29;15(21):3884. doi: 10.3390/foods15213884 (PMC13762881; doi:10.3390/foods15213884)
Supplement: Supplementary file 1 [file foods-15-03884-s001.zip › foods-4555866-supplementary.pdf]

Supplementary Material: Sensory Evaluation Questionnaires

Questionnaire S1. Preliminary Sensory Evaluation Questionnaire

SENSORY EVALUATION SHEET

Date: ______________	Panelist No.: ______________

Please examine and taste each sample, then assign a score from 1 to 9 for color, taste, and overall acceptability.
Samples will be presented in randomized order. Please rinse your mouth with water between samples.

9 = Like extremely	4 = Dislike slightly
8 = Like very much	3 = Dislike moderately
7 = Like moderately	2 = Dislike very much
6 = Like slightly	1 = Dislike extremely
5 = Neither like nor dislike	

Sample code	Color	Taste	Overall acceptability
129	____	____	____
274	____	____	____
518	____	____	____
693	____	____	____

Which of the three enriched samples did you prefer overall?

☐ 274 ☐ 518 ☐ 693

Optional comments:

____

____

Thank you for your participation.

Questionnaire S2. Consumer Acceptance Questionnaire for the Selected Breadstick Formulation

Date:	________________	Consumer No.:	______________
Gender:	________________	Age:	______________

Please rate each attribute using the following 5-point scale:

1 = Not at all satisfactory

2 = Slightly satisfactory

3 = Neither satisfactory nor unsatisfactory

4 = Quite satisfactory

5 = Very satisfactory

Attribute	Scale from 1 (not at all satisfactory) to 5 (very satisfactory)
Appearance and presentation	□ 1 □ 2 □ 3 □ 4 □ 5
Breadstick size	□ 1 □ 2 □ 3 □ 4 □ 5
Aroma (intensity and pleasantness)	□ 1 □ 2 □ 3 □ 4 □ 5
Crispness	□ 1 □ 2 □ 3 □ 4 □ 5
Overall taste	□ 1 □ 2 □ 3 □ 4 □ 5
Aftertaste	□ 1 □ 2 □ 3 □ 4 □ 5
Ease of chewing	□ 1 □ 2 □ 3 □ 4 □ 5
Overall liking	□ 1 □ 2 □ 3 □ 4 □ 5

Thank you for participating in this tasting and sensory evaluation activity.

Your opinion is essential for assessing product acceptance and for guiding possible improvements in the formulation and characteristics of the breadsticks. Your contribution provides valuable support for the development of foods that increasingly meet consumer preferences.
